# Receptor-binding domain of SARS-CoV-2 spike protein efficiently inhibits SARS-CoV-2 infection and attachment to mouse lung

**DOI:** 10.7150/ijbs.61320

**Published:** 2021-08-28

**Authors:** Hye Jin Shin, Keun Bon Ku, Hae Soo Kim, Hyun Woo Moon, Gi Uk Jeong, Insu Hwang, Gun Young Yoon, Sunhee Lee, Sumin Lee, Dae-Gyun Ahn, Kyun-Do Kim, Young-Chan Kwon, Bum-Tae Kim, Seong-Jun Kim, Chonsaeng Kim

**Affiliations:** Center for Convergent Research of Emerging Virus Infection, Korea Research Institute of Chemical Technology, Daejeon 34114, South Korea

**Keywords:** SARS-CoV-2, COVID-19, receptor-binding domain, anti-viral, therapeutics

## Abstract

COVID-19, caused by a novel coronavirus, SARS-CoV-2, poses a serious global threat. It was first reported in 2019 in China and has now dramatically spread across the world. It is crucial to develop therapeutics to mitigate severe disease and viral spread. The receptor-binding domains (RBDs) in the spike protein of SARS-CoV and MERS-CoV have shown anti-viral activity in previous reports suggesting that this domain has high potential for development as therapeutics. To evaluate the potential antiviral activity of recombinant SARS-CoV-2 RBD proteins, we determined the RBD residues of SARS-CoV-2 using a homology search with RBD of SARS-CoV. For efficient expression and purification, the signal peptide of spike protein was identified and used to generate constructs expressing recombinant RBD proteins. Highly purified RBD protein fused with the Fc domain of human IgG showed potent anti-viral efficacy, which was better than that of a protein fused with a histidine tag. Intranasally pre-administrated RBD protein also inhibited the attachment of SARS-COV-2 to mouse lungs. These findings indicate that RBD protein could be used for the prevention and treatment of SARS-CoV-2 infection.

## Introduction

The new coronavirus was first reported as a lethal pathogen, causing serious illness, pneumonia, and deaths, in December 2019. The novel coronavirus (CoV) is phylogenetically related to severe acute respiratory syndrome coronavirus (SARS-CoV) and has been named SARS-CoV-2 1. SARS-CoV-2 is a highly pathogenic human CoV that has dramatically spread across the world, posing serious global public health threats. The global pandemic situations urgently call for drugs 2. Coronaviruses are enveloped, single-stranded positive-sense RNA viruses including Middle East respiratory syndrome coronavirus (MERS-CoV), SARS-CoV and SARS-CoV-2, which have been identified as zoonotic outbreaks of beta-CoV 2-4. All CoVs have four structural proteins, including spike (S), envelope (E), membrane (M), and nucleocapsid (N) proteins which are necessary for completion of the viral replication cycle 5, 6. CoV uses the S protein for binding with host receptors to mediate membrane fusion and virus entry into host cells. The receptor-binding domain (RBD) in the S1 subunit of the S protein mediates initial attachment to a host receptor and then fusion of the viral and host membranes is mediated through the S2 subunit 7-9. The RBDs of SARS-CoV-2 and MERS-CoV recognize different receptors, angiotensin-converting enzyme 2 (ACE2) and dipeptidyl peptidase 4 (DPP4), respectively 10, 11. Previous reports have shown that RBDs of SARS-CoV or MERS-CoV efficiently inhibit virus infection by binding to its own receptor 12, 13. These results suggest that the RBD has high potential for development as a therapeutic inhibitor of viral entry 14, 15.

In this study, to test the antiviral activity of recombinant RBD protein of SARS-CoV-2 against this virus, the RBD region was determined using a homology search, compared with the RBD region of SARS-CoV. The signal peptide in the spike protein of SARS-CoV-2 was also identified to facilitate recombinant protein expression and purification. Highly purified recombinant RBD proteins from SARS-CoV-2 were produced by human cells. These proteins showed potent antiviral effects against SARS-CoV-2 infection *in vitro*. Intranasally applied proteins inhibited the SARS-CoV-2 attachment to mouse lungs. Recombinant RBD proteins thus have the potential to be developed as therapeutics and prophylactics against SARS-CoV-2 infection.

## Materials and Methods

### Viruses and cells

Vero cells (CCL-81) and HEK-293 cells (CRL-1573) were purchased from the American Type Culture Collection (ATCC) and were maintained in Dulbecco's modified Eagle medium supplemented with 10% fetal bovine serum (both HyClone, San Angelo, TX, USA). The cell lines were incubated in a 5% CO_2_ humidified atmosphere at 37°C. SARS-CoV-2 (BetaCoV/Korea/KCDC03/2020, NCCP43326) was kindly provided by the Korean Centers for Disease Control and Prevention 16. SARS-CoV-2 was propagated and prepared in Vero cells. All experiments using SARS-CoV-2 were handled in an enhanced biosafety level 3 (BSL-3) containment laboratory as approved by the Korean Center for Disease Control and Prevention.

### Antibodies

Primary antibodies used in the study include the following: mouse monoclonal anti-β-actin (Cell Signaling), mouse monoclonal anti J2 double stranded RNA (dsRNA; English and Scientific Consulting Kft, Szirák, Hungary), rabbit polyclonal anti-SARS-CoV-2 spike (Sino Biological, #40591-V08H) and rabbit polyclonal anti-MERS spike (Sino Biological, #40069-RP02). The secondary antibodies used for immunofluorescence were Alexa Fluor 488 goat anti-mouse. The secondary antibodies used for western blot analyses were HRP-conjugated anti-rabbit IgG (Invitrogen, Waltham, MA, USA) and HRP-conjugated anti-human IgG (Invitrogen).

### Mouse experiments and preparation of lung tissue samples

All animal experiments were reviewed and approved by the Institutional Animal Care and Use Committee (IACUC) of the Korea Research Institute of Chemical Technology (2020-8B-07-01). Nine 6-week-old female C57BL/6 mice were evenly divided into three groups and housed in the BSL-3 animal facility. Mice from each group were lightly euthanized with isoflurane and administered PBS, SARS2-RBD-Fc, or MERS-RBD-Fc protein sample (15 mg/kg per mouse) intranasally. SARS-CoV-2 was injected into each mouse after 1 h via the intranasal route. Five hours after the first protein injection, mouse lung samples were washed with excess PBS through the trachea using a syringe and harvested to test the inhibition of SARS-CoV-2 attachment. Tissue samples from the lung were homogenized in cold phosphate-buffered saline (PBS) solution with a FastPrep-24 homogenizer (MP Biomedicals) for 5 cycles (20 s on/ 20 s off). Three additional freeze-thaw cycles were performed at -80°C, and cell debris was removed by centrifugation at 13000rpm for 1 min to measure the viral RNA in the lung.

### Real-time qRT-PCR

To analyze the expression levels of intracellular genes and SARS-CoV-2 viral genes, total RNA was extracted from mouse lung samples using the RNeasy Mini Kit (Qiagen, Hilden, Germany). The SARS-CoV-2 RNA copy numbers were determined by real-time qRT-PCR using the One Step PrimeScript III RT-PCR Kit (Takara). The following primer sets were used for RT-PCR: SARS-CoV-2_N2 forward, 5′-TTACAAACATTG GCCGCAAA; SARS-CoV-2_N2 reverse 5′-GCGCGACATTCCGAAGAA; SARS-CoV-2_N2 probe 5′-FAM-ACAATTTGCCCCCAGCGCTTCAG-BHQ1; β-actin forward, 5′-GATTACTGCTCTGGCTCCTAG; β-actin reverse, 5′-GACTCATCGTACTCCTGCTTG, β-actin probe; 5′-/56-FAM/CTGGCCTCA/ZEN/CTGTCCACCTTCC/3IABkFQ Real-time qPCR was conducted using the QuantStudio3 Real-Time PCR System (Applied Biosystems, Waltham, MA, USA).

### Antiviral activity and cell toxicity determination

To evaluate antiviral activity and cell toxicity with recombinant RBD proteins, 2 × 10^4^ Vero cells per well were seeded in 96-well plates 1 day before infection. Serially diluted recombinant RBD proteins (100 μg/mL to 0.16 μg/mL) were added to the cells 1 h prior to infection. The cells were infected with SARS-CoV-2 at a multiplicity of infection of 3 in a BSL3 facility. At 1 day post-infection, cells were fixed with 4% paraformaldehyde and permeabilized with 0.1% triton X-100 in phosphate-buffered saline. The cells were immunostained with an anti-dsRNA antibody and AF488-conjugated anti-mouse secondary antibody. Nuclei were counterstained with Hoechst 33342 (Thermo Fisher Scientific, Waltham, MA, USA). Image acquisition and analysis were performed as previously described 17. Viral infection was quantified by dividing the number of cells stained with anti-dsRNA antibody by the total number of cells (obtained by counting the nuclei). Infection rate standards were obtained from mock-infected cells (0%) and SARS-CoV-2 only-infected cells (100%). Infection rates in recombinant RBD protein-administered cells were calculated based on these standards. The recombinant RBD protein antiviral activity was determined from dose-response curves; half maximal inhibitory concentration (IC_50_) values were calculated using Prism v8 software (GraphPad Software). To determine the cell toxicity (CC_50_), similar experiments were performed without addition of the virus; cell viability was measured using MTT solution. CC_50_ values were calculated using Prism v8 software. The selectivity index (SI) was calculated as CC_50_/IC_50_.

### Protein expression and purification

The expression and purification of MERS-RBD-His and MERS-RBD-Fc were performed as previously described 18. To express SARS2-RBD-His, a codon-optimized sequence was synthesized encoding the signal sequence (residues 1-16) and RBD (residues 319-541) of SARS-CoV-2 spike protein and a 6×His tag. To express SARS-RBD-Fc, the sequence encoding human IgG Fc was replaced instead of the 6×His tag. The synthesized DNA was subcloned into a mammalian expression vector. Recombinant proteins were expressed using Expi293F cells and a transient expression system (Thermofisher Scientific) following the manufacturer's instructions. SARS2-RBD-His protein was purified from the supernatant using Ni-NTA affinity column (Qiagen). SARS2-RBD-Fc protein was purified from the supernatant using protein G column (GE Healthcare). Purified protein was dialyzed against PBS and concentrated using an Amicon Ultra-15 centrifugal filter (Millipore, #UFC901024). Proteins were analyzed by SDS-PAGE with Coomassie blue staining and western blotting using the indicated antibodies.

## Results

### Determination of RBD and the signal peptide of the spike protein of SARS-CoV-2 and expression of recombinant RBD proteins

**Figure 1:**
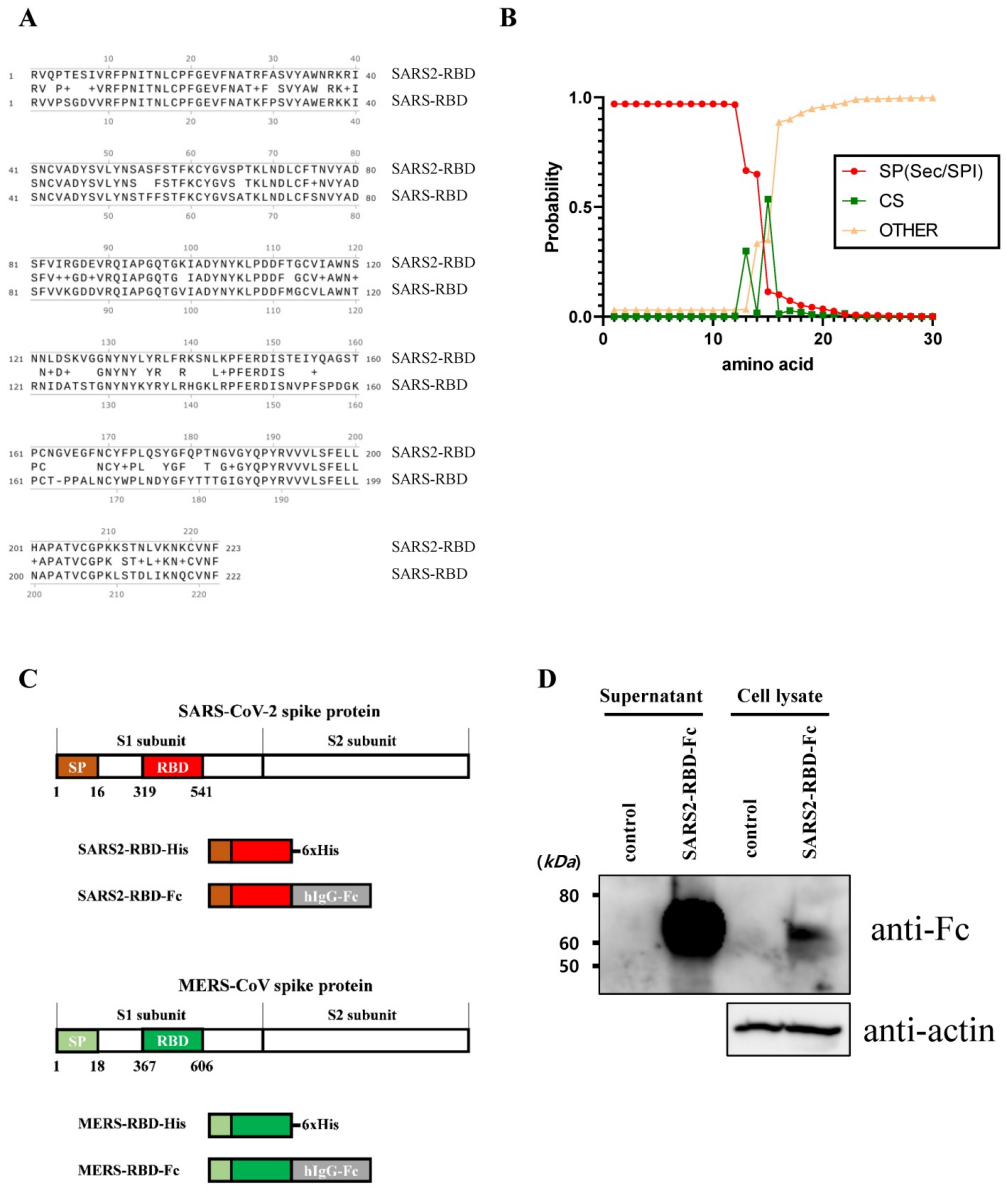
** Identification of receptor-binding domain (RBD) and signal sequence in spike protein of SARS-CoV-2.** (A) Sequence alignment of RBDs of SARS-CoV-2 and SARS-CoV. Conserved residues are indicated by amino acid in the middle line. + represents similar residues. (B) Analysis result of the signal sequence of SARS-CoV-2 spike protein using the SignalP program. (C) Schematic diagram of constructs expressing the RBD proteins of SARS-CoV-2 and MERS-CoV. (D) The SARS2-RBD-Fc construct was transfected into HEK-293 cells for expression tests. Cells (Cell lysate) and culture supernatant (supernatant) were analyzed by western blotting using an anti-human Fc antibody. Actin was used as a loading control for cell lysates.

**Figure 2:**
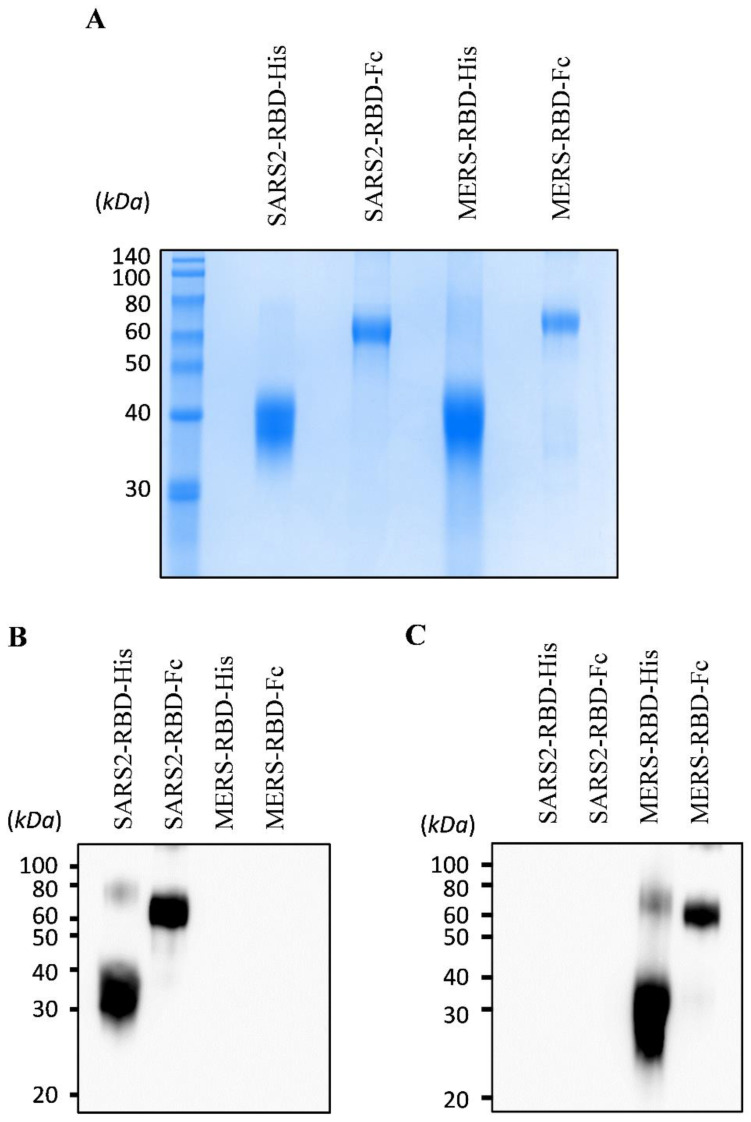
** Expression and purification of recombinant receptor-binding domain (RBD) proteins of SARS-CoV-2 and MERS-CoV.** Purified proteins were analyzed by Coomassie staining using SDS-PAGE (A). The identity of the purified protein was confirmed by immunoblotting using an anti-SARS-CoV-2 spike antibody (B) and an anti-MERS-CoV spike antibody (C).

To express and purify the RBD protein of SARS-CoV-2, we first determined the RBD and signal peptide of the spike protein of this virus. The RBD region of SARS-CoV-2 was identified at residues 319-541 of the S protein (Fig. 1A) using a homology search with RBD of SARS-CoV 8. The signal peptide sequence of the SARS-CoV-2 S protein was predicted using the SignalP web server (http://www.cbs.dtu.dk/services/SignalP/) (Fig. 1B). This prediction showed that the spike protein of SARS-CoV-2 has a Sec signal peptide (Sec/SPI) and the probability of a cleavage site was highest between amino acids 15 and 16 (CS). After amino acid 16, the probability that the sequence does not have any kind of signal peptide (OTHER) increased to 1.0, which means that there is no signal peptide after amino acid 16. This signal peptide could induce the secretion of recombinant protein into the culture supernatant for convenient protein purification and post-translational modifications such as glycosylation, similar to the spike protein on the naïve virus. We then generated a recombinant RBD expression construct, fusing the genes encoding residues of the signal peptide (1-16) and RBD (319-541) of the SARS-CoV-2 spike protein with a 6×His tag (SARS2-RBD-His) or Fc fragment of human IgG1 (SARS2-RBD-Fc), as shown in Fig. 1C. The Fc fragment can improve the solubility and stability of the fusion protein and is expressed as a homodimer 19. The antiviral activity of the RBD-Fc fusion protein was compared with that of RBD with a 6×His tag to investigate the functional advantage of the Fc fragment. To determine the specificity of the RBD protein, similar constructs expressing RBD of MERS-CoV were generated with 6×His tag (MERS-RBD-His) or Fc fragment of human IgG1 (MERS-RBD-Fc) (Fig. 1C). The expression and secretion of RBD proteins were tested in HEK-293 cells based on transient transfection of the SARS2-RBD-Fc construct. SARS2-RBD-Fc was highly expressed and secreted into the culture supernatant (Fig. 1D). Each protein was expressed efficiently in human cells and could be readily purified from the culture supernatant using a Ni-NTA affinity column or protein G column. Four types of purified recombinant proteins were analyzed by Coomassie staining (Fig. 2A) and their identities were confirmed by western blotting with anti-SARS-CoV-2 spike (Fig. 2B) and anti-MERS-CoV spike (Fig. 2C) antibodies.

### The potent antiviral activity of recombinant SARS2 RBD-Fc protein *in vitro*

**Figure 3:**
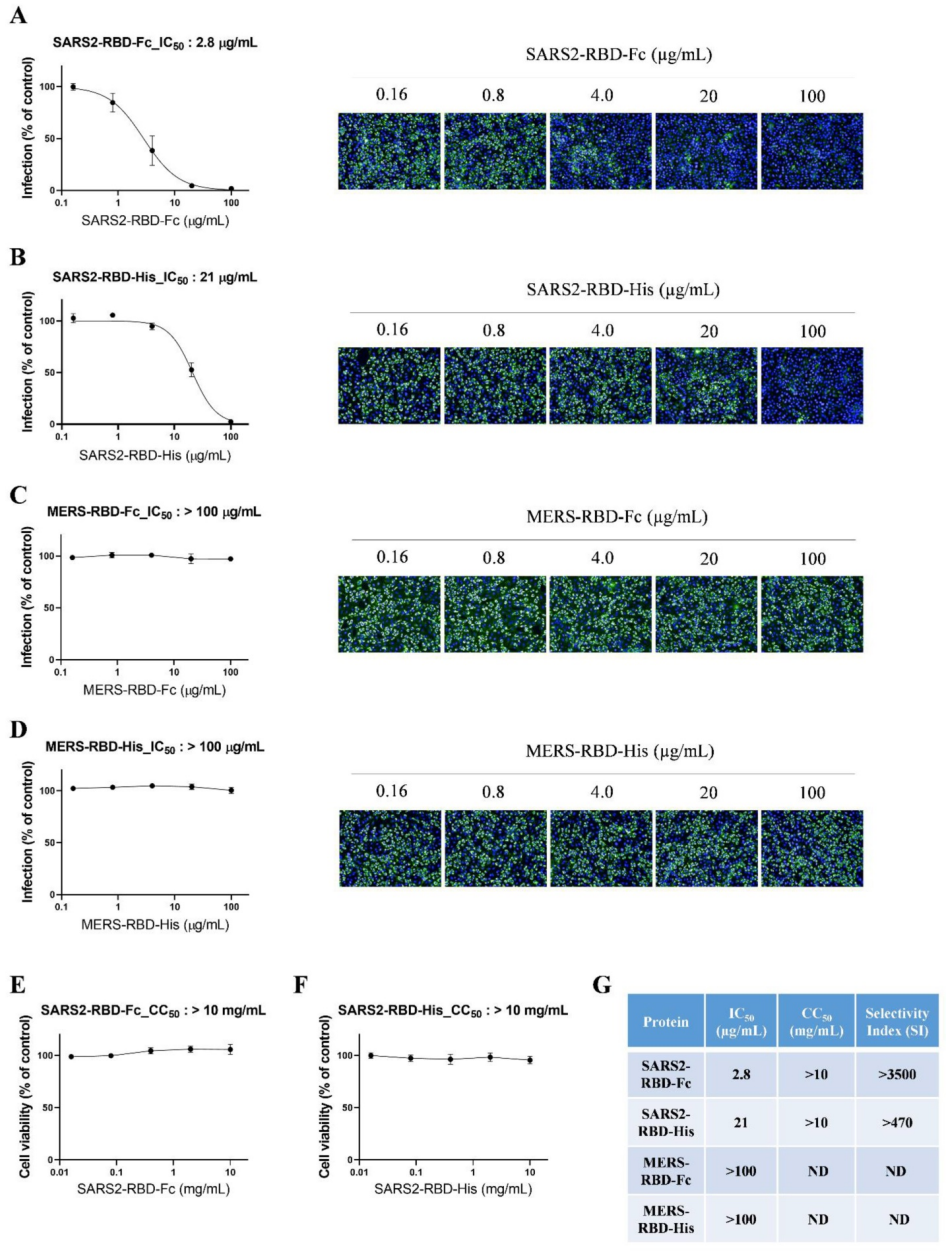
** Potent antiviral activity of SARS2-RBD-Fc protein against cellular infection by SARS-CoV-2**. (A) Vero cells were pre-treated with SARS2-RBD-Fc protein at the indicated concentrations and infected with SARS-CoV-2. After 24 h, cells were fixed and stained with an anti-dsRNA antibody. Infected cells were visualized with an Alexa Fluor 488-conjugated secondary antibody (green). Nuclei were stained with Hoechst 33342 (blue). Virus infection rates were determined by counting the total cells (blue) and infected cells (green), and antiviral activity (IC_50_) was determined from a dose-response curve. A similar experiment as performed in (A) was performed using SARS2-RBD-His (B), MERS-RBD-Fc (C), and MERS-RBD-His (D). To determine the cell toxicity (CC_50_), SARS2-RBD-Fc protein (E) and SARS2-RBD-His (F) were administered to cells at the indicated concentrations. After 24 h, cell viability was measured using an MTT assay. (G) Antiviral activity (IC_50_), cell toxicity (CC_50_), and selectivity index (SI) of four recombinant proteins are summarized. ND, not determined.

To evaluate the potential antiviral activity of recombinant SARS-CoV-2 RBD proteins, we performed an antiviral assay based on immunofluorescent staining using anti-dsRNA antibodies that detect dsRNA in cells infected with diverse positive-stranded RNA viruses, such as SARS-CoV 20. We incubated serially diluted SARS2-RBD-Fc, SARS2-RBD-His, MERS-RBD-Fc, or MERS-RBD-His proteins (0.16-100 μg/ml) with Vero cells, followed by infection with SARS-CoV-2. SARS-CoV-2-infected cells showed a green fluorescent signal, which gradually decreased in a dose-dependent manner following treatment with recombinant SARS2-RBD-Fc or SARS2-RBD-His proteins (Fig. 3A and 3B). Both recombinant SARS2-RBD-Fc and SARS2-RBD-His proteins showed antiviral activity against SARS-CoV-2 with IC_50_ values of 2.8 and 21 μg/mL, respectively (Fig. 3A and 3B). SARS2-RBD-Fc protein showed 7.5-fold better antiviral effects than SARS2-RBD-His.

In addition, MERS-RBD-Fc or MERS-RBD-His did not inhibit the infection of SARS-CoV-2 in cells (Fig. 3C and 3D). Only RBD proteins from SARS-COV-2 were specific for SARS-CoV-2. The cell toxicity of these proteins was assessed in parallel with their antiviral efficacy over a broader concentration range (0.016-10 mg/mL). No significant cell toxicity was observed at up to 10 mg/mL with SARS2-RBD-Fc and SARS2-RBD-His treatment (Fig. 3E and 3F). The IC_50_ values are summarized in Fig. 3G, which also shows the cell toxicity (CC_50_) and SI values. Notably, SARS2-RBD-Fc protein was found to have a high selectivity index, with a CC_50_ value 3500-fold higher than the IC_50_ value. Our results suggest that recombinant SARS2-RBD-Fc proteins have potent antiviral activity against SARS-CoV-2 and could be considered a promising therapeutic agent against SARS-CoV-2 infection.

### Intranasally applied SARS2-RBD-Fc protein results in strong inhibition of SARS-CoV-2 attachment to mouse lung

To test the antiviral activity of RBD proteins *in vivo*, an animal model of SARS-CoV-2 is required. Although transgenic mice that overexpress human ACE2 were developed as an animal model, they are currently limited in use. One group reported that mouse ACE2 barely supports entry of the pseudotyped SARS-CoV-2 virus 21.

**Figure 4:**
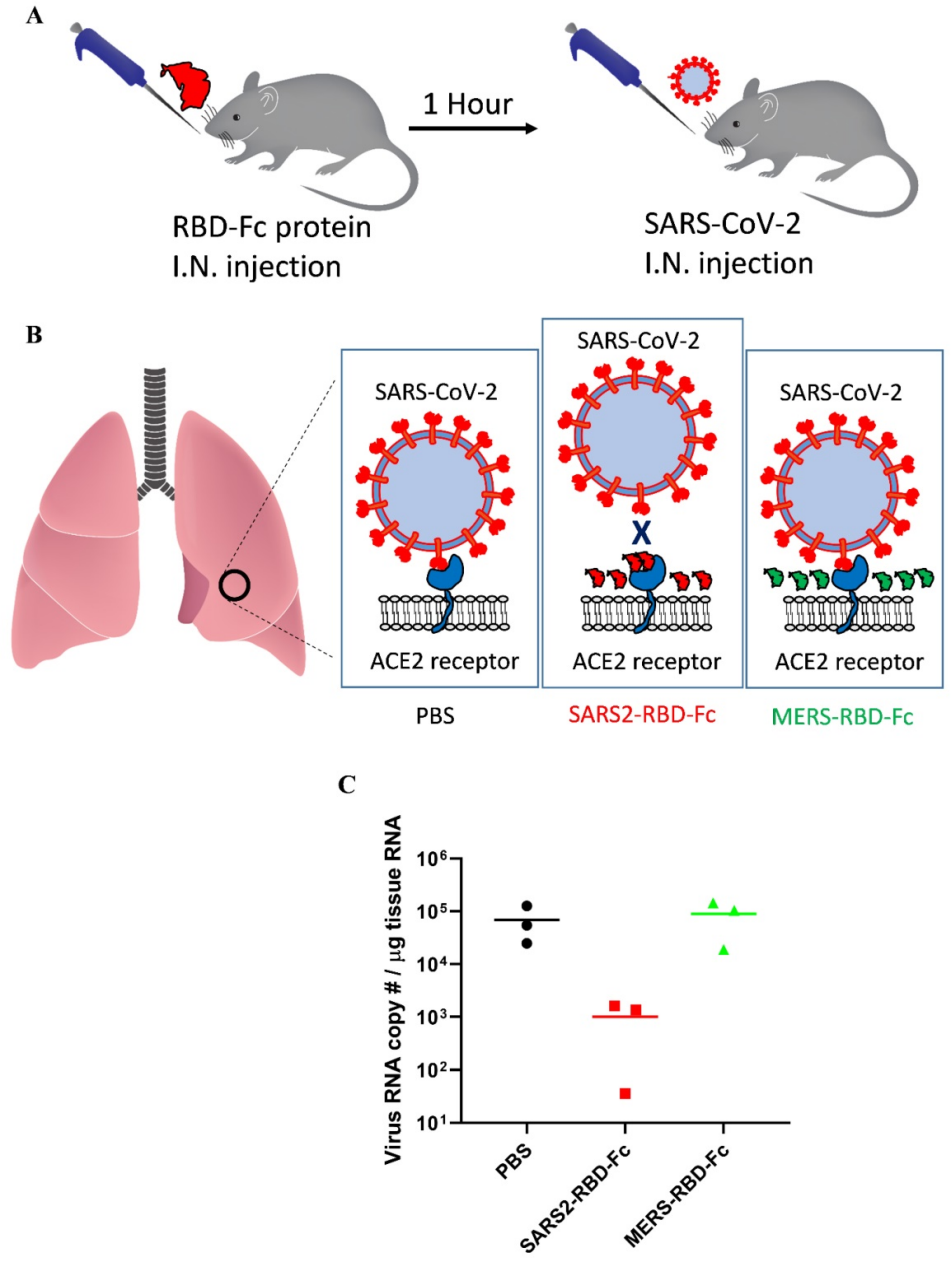
** Potent inhibition of SARS-CoV-2 attachment to mouse lungs via intranasally administered SARS2-RBD-Fc protein.** (A) Schematic diagram of mouse experiment to test the inhibition of virus attachment. (B) A potential explanation showing the inhibition of virus binding to mouse lung by SARS2-RBD-Fc. PBS and MERS-RBD-Fc were used as negative controls. (C) Tissue RNA isolated from mouse lung was used for the analysis of SARS-CoV-2 levels using PCR primers specific to the SARS-CoV-2 nucleocapsid gene.

For SARS-CoV, mouse ACE2 binds the virus less efficiently and supports decreased virus infection 22. These reports suggested that mouse ACE2 could support SARS-CoV-2 attachment with reduced activity and that we could test the inhibition of virus attachment by SARS2-RBD-Fc protein using wild-type mice only expressing mouse ACE2. As we used an RBD sequence that was the same as that of the virus, the RBD protein could more efficiently bind human ACE2, and the results confirmed in the experiment using mouse ACE2 will be applicable to human ACE2 as well. SARS2-RBD-Fc was tested in mouse lungs to examine the inhibition of virus attachment *in vivo*. Mice were evenly divided into three groups and pre-administered PBS, SARS2-RBD-Fc, or MERS-RBD-Fc protein sample intranasally, and then, SARS-CoV-2 was injected into each mouse after 1 h via the intranasal route (Fig. 4A). Pre-administration of SARS2-RBD-Fc could occupy mouse ACE2 and inhibit the attachment of SARS-CoV-2 (Fig. 4B). PBS and MERS-RBD-Fc served as negative controls (Fig. 4B). Five hours after the first protein injection, mouse lung samples were washed with excess PBS and harvested to test the inhibition of SARS-CoV-2 attachment. To analyze the copy number of SARS-CoV-2 RNA, total RNA was extracted from mouse lung samples and the titer of viral RNA was determined by real-time qRT-PCR (Fig. 4C). Notably, treatment with SARS2-RBD-Fc resulted in a >100-fold decrease in the copy number of SARS-CoV-2 RNA in mouse lung compared to that with PBS or MERS-RBD-Fc treatment, reflecting the fact that SARS2-RBD-Fc effectively blocks the binding of SARS-CoV2 to the ACE2 receptor in the mouse lung (Fig. 4C). We confirmed that the attachment of SARS-CoV-2 was significantly prevented by the pre-administration of SARS2-RBD-Fc *in vivo*.

## Discussion

As the current COVID-19 pandemic is a severe threat globally, many attempts are being made worldwide to develop therapeutics and vaccines. Over 10 different COVID-19 vaccines have been administered worldwide, remdesivir is an FDA-approved COVID-19 drug, and several monoclonal antibodies have been approved for use through emergency use authorization. In this study, our results suggested that recombinant SARS2-RBD-Fc protein has potent antiviral activity against SARS-CoV-2. While preparing this manuscript, another group reported that the RBD protein of SARS-CoV-2 inhibits virus binding to ACE2-expressing cells 23. They showed that SARS-CoV-2 RBD protein binds to human ACE2 and inhibits SARS-CoV-2 pseudovirus entry with an IC_50_ of 1.35 μg/mL. This result was in good agreement with our antiviral activity data for live SARS-CoV-2 infection, which was associated with an IC_50_ of 2.8 μg/mL. Their identified RBD at residues 331 to 524 of the S protein was relatively small compared to our RBD protein at residues 317-541. Small additional regions of our RBD at residues 317-330 and 525-541 did not have a significant effect. Although we and Tai et al. showed good antiviral activity of RBD protein, our results were different in a few points, such as the use of live SARS-CoV-2 instead of pseudotyped virus and the demonstration of in vivo antiviral effect.

We also determined that intranasally administrated SARS2-RBD-Fc protein decreases the attachment of SARS-CoV-2 to mouse lungs, whereas MERS-RBD-Fc treatment did not show any marked effect on SARS-CoV-2 binding. Mouse ACE2 has low sensitivity to SARS-CoV compared to human ACE2, but can still bind the virus 22. Another report determined that mouse ACE2 barely supports the entry of psudotyped SARS-CoV-2 21. Using wild-type mice, we showed that the attachment of SARS-CoV-2 to mouse lungs was detectable using qRT-PCR. Currently, there is no perfect animal model that directly reproduces the pathology seen in humans 24. Transgenic mice expressing human ACE2 are limited for use. Our results suggest that wild-type mice could be an alternative *in vivo* model to test inhibitors of virus-ACE2 interactions, such as neutralizing antibodies, chemicals, and therapeutic proteins.

The recombinant SARS2-RBD-Fc protein fused with the Fc domain of human IgG showed better antiviral activity than the RBD protein. Notably, SARS2-RBD-Fc effectively blocked SARS-CoV-2 infection with an IC_50_ of 2.8 μg/mL, which was 7.5-fold higher than that of SARS2-RBD-His. The presence of the Fc domain of the recombinant protein can improve the stability and solubility of proteins 25. A recent report described that the dimeric form of RBD protein significantly increases immunogenicity compared to that with the monomer form of RBD 26, and homodimer formation by the Fc domain could be a reason for the enhanced antiviral activity. The crystal structure revealed that the form of the RBD dimer fully exposes receptor-binding motifs and enhances stable expression, which results in potent antiviral efficacy, compared to that with the conventional monomeric form 26.

A primary site of SARS- CoV-2 infection appears to be the nasal epithelium, upper airways, and lungs, and then, the virus spreads to other human tissues. In the upper respiratory tract, SARS-CoV-2 induces efficient replication and rapid transmission between hosts 27. Therefore, recombinant RBD protein might serve as a viral attachment inhibitor against SARS-CoV-2 infection through its injection or nasal spray into the respiratory tract of the host. In the context of the current COVID-19 pandemic, our study suggests that the development of SARS-CoV-2 RBD-based drugs is a promising approach to prevent viral attachment or membrane fusion and could be used for the treatment of coronavirus-related diseases.
